# Human Umbilical Cord Mesenchymal Stem Cell-Derived Microvesicles Could Induce Apoptosis and Autophagy in Acute Myeloid Leukemia

**DOI:** 10.61186/ibj.27.5.247

**Published:** 2023-06-04

**Authors:** Mohammad Khani-Eshratabadi, Seyed Hadi Mousavi, Morteza Zarrabi, Jamal Motallebzadeh Khanmiri, Zahra Zeinali Bardar

**Affiliations:** 1 Department of Hematology and Blood Transfusion Sciences, School of Allied Medical Sciences, Tehran University of Medical Sciences, Tehran, Iran;; 2 Kashmar School of Medical Sciences, Mashhad University of Medical Sciences, Mashhad, Iran;; 3 Department of Regenerative Medicine, Cell Science Research Center, Royan Institute for Stem Cell Biology and Technology, ACECR, Tehran, Iran;; 4 Student Research Committee, Mashhad University of Medical Sciences, Mashhad, Iran

**Keywords:** Acute myeloblastic leukemia, Apoptosis, Autophagy, Mesenchymal stem cells

## Abstract

**Background:**

Microvesicles have been identified as candidate biomarkers for treating AML. This study investigated the effects of hUCMSC-derived MVs on apoptosis and autophagy in the KG-1 leukemic cell line.

**Methods:**

The hUCMSCs were cultured and characterized by flow cytometry. MVs were isolated by ultracentrifugation, and the concentration was determined using the Bradford method. The characteristics of MVs were confirmed by TEM, flow cytometry, and DLS methods. KG-1 cells were treated with the desired concentrations of MVs for 24 h. The apoptosis induction and ROS production were evaluated using flow cytometry. RT-PCR was performed to evaluate apoptosis- and autophagy-related genes expression.

**Results:**

Following tretment of KG-1 cells with 25, 50, and 100 μg/ml concentrations of MVs, the apoptosis rates were 47.85%, 47.15%, and 51.35% (p < 0.0001), and the autophagy-induced ROS levels were 73.9% (p < 0.0002), 84.8% (p < 0.0001), and 85.4% (p < 0.0001), respectively. *BAX *and *ATG7* gene expression increased significantly at all concentrations compared to the control, and this level was higher at 50 μg/ml than that of the other concentrations. In addition, *LC3* and *Beclin 1* expression increased significantly in a concentration-dependen manner. Conversely, *BCL2* expression decreased compared to the control.

**Conclusion:**

Our findings indicate that hUCMSC-MVs could induce cell death pathways of autophagy and apoptosis in the KG-1 cell lines and exert potent antiproliferative and proapoptotic effects on KG-1 cells in vitro. Therefore, hUCMSC-MVs may be a potential approach for cancer therapy as a novel cell-to-cell communication strategy.

## INTRODUCTION

Leukemic cells can infiltrate into various tissues, including liver, spleen, gums, skin, and the central nervous system^[^^1^^]^. In patients with AML, bone marrow failure and abnormal maturation of normal blood cells lead to complications such as thrombocytopenia-induced bleeding, severe anemia, and infection due to neutropenia^[^^2^^,^^3^^]^. Previous studies have revealed that AML is associated with genetic mutations, epigenetic alterations in vital functional genes, exposure to carcinogenic agents, chemotherapy, and radiation, as well as unhealthy lifestyle^[^^4^^,^^5^^]^. High relapse rates, bone marrow transplantation rejections, multidrug resistance against various common chemotherapies, poor prognosis of some patients, and high mortality rates of AML, even with great advances in chemotherapy and stem cell transplantation, make AML responsible for many annual cancer-related deaths^[^^6^^-^^8^^]^. Therefore, new treatments of AML are urgently required to establish highly efficient therapeutic strategies.

Currently, paracrine factors and bioactive substrates released from MSCs are potential therapeutic agents for hematological disorders, particularly AML^[^^9^^-^^11^^]^. MSCs are capable of self-renewal and multilineage differentiation and often derived from varying tissue sources, e.g. bone marrow, umbilical cord, and adipose tissue. hUCMSCs are favorable because of the noninvasive and stress-free isolation procedure^[^^12^^,^^13^^]^. The hUCMSCs affect target cells via direct cell-to-cell contact by secreting a wide range of MVs^[^^14^^]^. MVs are small biological and spherical membrane fragments with a size ranging from 100 to 1000 nm and contain different cytokines, cytoplasmic proteins, membrane proteins (such as receptors), antitumor microRNAs, and noncoding RNAs^[^^11^^,^^15^^]^. MSC-MVs have been shown to inhibit the growth and proliferation of leukemic cells. They also indicate proapoptotic and autophagy-stimulating activity, owing to several types of growth factors and proapoptotic molecules^[^^11^^,^^14^^,^^16^^-^^18^^]^. These tumor-suppressive properties of MVs can induce anti-tumor activity^[^^19^^-^^22^^]^. 

Considering the above-mentioned data, hUCMSCs inhibit leukemic cell growth and proliferation. We hypothesize that hUCMSC-MVs may suppress the proliferation of the KG-1 cell line (human acute myeloid leukemia) by inducing apoptosis and autophagy.

## MATERIALS AND METHODS

**Cell culture and identification**

The hUCMSCs were purchased from Royan Institute (Tehran, Iran) and seeded into a culture flask in a growth medium containing DMEM (Gibco, CA, USA) supplemented with 10% FBS (Merck, Darmstadt, Germany) and 1% penicillin/streptomycin (Gibco) in 95% humidity and 5% CO_2_ at 37 °C. Every 72 h, the culture medium was changed. Once the adherent cells reached 80% confluency, they were passaged using 0.5% trypsin-EDTA (Gibco). After reaching 80-90% density at the sixth passage, the cells were seeded into six-well plates at 1.3 × 10^5^ cells per well. Cell marker expression was detected by flow cytometry using antibodies against CD73, CD34/CD45, CD90, and CD105 (all from BD Biosciences, USA) conjugated with FITC or phycoerythrin. The KG-1 leukemic cell line was procured from the Pasteur Institute of Iran (Tehran) and cultured in RPMI (Gibco) supplemented with 10% FBS and 1% penicillin/streptomycin in 85% humidity and 5% CO_2_ at 37 °C. The medium was changed every two days. The cell count and the viable cells were counted by trypan blue staining. Cells were counted using an automated Countess Cell Counter (Invitrogen, CA, USA). Living cells remained unstained, whereas dead cells turned blue in color.

**Isolation of hUCMSC-MVs**

MVs were isolated from the hUCMSC supernatants. Briefly, hUCMSCs were cultured in DMEM supplemented with 10% FBS and incubated in an incubator with 5% CO_2_ and 95% humidity. When the cells reached 90% confluence at the sixth passage, the superficial layer (liquid) was removed for the next step. The viability of the cells was >92% after 72 h. The cell-free supernatant was centrifuged in two steps: 2000 ×g for 20 min for cell debris removal and 100,000 ×g in an SW41 swing rotor (Beckman Coulter Optima L-80K ultracentrifuge; Beckman Coulter, Fullerton, CA, USA) at 4 °C for 2 h. Finally, the MVs were stored at -70 °C until further use.

**Characterization of hUCMSC-MVs by TEM**

MVs were first fixed in 2.5% glutaraldehyde in PBS for 2 h and then washed, ultracentrifuged, and suspended in 100 ml of PBS. Afterwards, 20 µl of MVs was loaded onto a formvar/carbon-coated grid, negatively stained with 3% aqueous phosphotungstic acid for 1 min and identified by TEM. (Hitachi, H-7650, Japan).

**Characterization of hUCMSC-MVs by DLS**

The particle sizes of the hUCMSC-MVs were analyzed by DLS. For particle size determination, the pellet was dissolved in PBS and analyzed by DLS.

**Measurement of protein content of hUCMSC-MVs**

To determine the amount of the isolated MVs, the protein content of hUCMSC-MVs was measured using the Bradford method following the manufacturer’s instructions.

**Co-culture of KG-1 cells with hUCMSC-MVs**

The human AML cell line KG-1 (1 × 10^5 ^cells/well) was seeded in 24-well plates in RPMI 1640 medium containing 10% FBS. Next, MVs were added to each well at the concentrations of 25, 50, and 100 μg/ml; each concentration was performed in triplicate. The plates were then incubated in an incubator for 24 h. KG-1 cells cultured in a fresh RPMI-1640 medium without hUCMSC-MVs were used as control.

**Apoptosis evaluation using annexin V/PI staining**

Cell apoptosis was measured using annexin V-FITC/PI staining (BioLegend, USA) and flow cytometry to evaluate the effect of hUCMSC-MVs on KG-1 cells. Briefly, the cells (1 × 10^5^) were treated with 25, 50, and 100 μg/ml of MVs in RPMI-1640 medium for 24 h (control: KG1 cells in fresh RPMI-1640 without MVs). The cells were harvested, washed twice with PBS and stained by Annexin V-FITC/PI in the dark at room temperature for 15 min and analyzed using a BD FACSCalibur flow cytometer (BD Biosciences).

**ROS assay**

The intracellular accumulation of ROS was assessed to investigate autophagy. ROS was identified using oxidized DCFH_2_-DA by flow cytometry. KG-1 cells were treated with three concentrations (25, 50, and 100 μg/ml) of MVs (control: KG1 cells in fresh RPMI-1640 without MVs) for 24 h. The cell pellets were resuspended in 0.3 ml of PBS containing 5 µM of DCFH_2_-DA and incubated in the dark at 37 °C for 20 min. The cells were then centrifuged at 2,000 ×g for 2 min and resuspended in 300 µl of PBS. Finally, the fluorescence intensity was measured using a BD FACSCalibur flow cytometer (BD Biosciences).

**Evaluation of apoptotic and autophagy gene expression**

To evaluate the effect of MVs on the induction of apoptosis and autophagy in KG-1 cells, the expression of *BCL2*, *BAX*, *Beclin 1*, *LC3*, and *ATG7* was evaluated by real-time PCR. Total RNA was extracted from the cultured cells 24 h after treatment, according to the *TRIzol* manufacturer’s protocol (Invitrogen), and then stored at -80 °C. RNA content was quantified using Nanodrop 2000c (Thermo Fisher Scientific, CA, USA). Complementary DNA was synthesized using PrimeScript First Strand cDNA Synthesis Kit (Thermo Fisher Scientific) following the manufacturer’s instructions and stored at -20 °C. Quantitative real-time PCR was carried out using the SYBR Green PCR Master Mix (Applied Biosystems, USA) in a lightCycler 360 Real-Time PCR Detection System (Roche, USA) using primers listed in Table 1. *GAPDH*, one of the most common housekeeping genes, was used in this study to compare the gene expression data.

**Statistical analysis**

Statistical analysis was performed using GraphPad Software (GraphPad Software, La Jolla, California, USA) and one-way analysis of variance (ANOVA). All experiments performed herein were replicated three times. P value less than 0.05 was considered statistically significant.

**Table 1:** Oligonucleotide primers used for RT-qPCR

**Gene**	**Primer sequences (5** **'** **3** **')**	**Product ** **length (kb)**
*BCL2*	F: CGACCACTAATTGCCAAGC R: TCCATCCGTCTGCTCTTCA	119
*BAX* *(Variant gamma)*	F: CTTCTGGAGCAGGTCACAGT R: AAGGTCACAGTGAGGTCAGG	146
*ATG7*	F: ATTGCTGCATCAAGAAACCC R: GATGGAGAGCTCCTCAGCA	121
*Beclin 1*	F: GGACACTCAGCTCAACGTCA R: AGCCTGGACCTTCTCGAGAT	208
*LC3*	F: CATGAGCGAGTTGGTCAAGAT R: TCGTCTTTCTCCTGCTCGTAG	138
*GAPDH*	F: CAACTACATGGTTTACATGTTCCAA R: CAGCCTTCTCCATGGTGGT	206

Data were presented as mean ± SD. Confidence limits were considered 95% in all tests.

## RESULTS

**Isolation and** **verification** **of hUCMSCs**

**Fig. 1:**
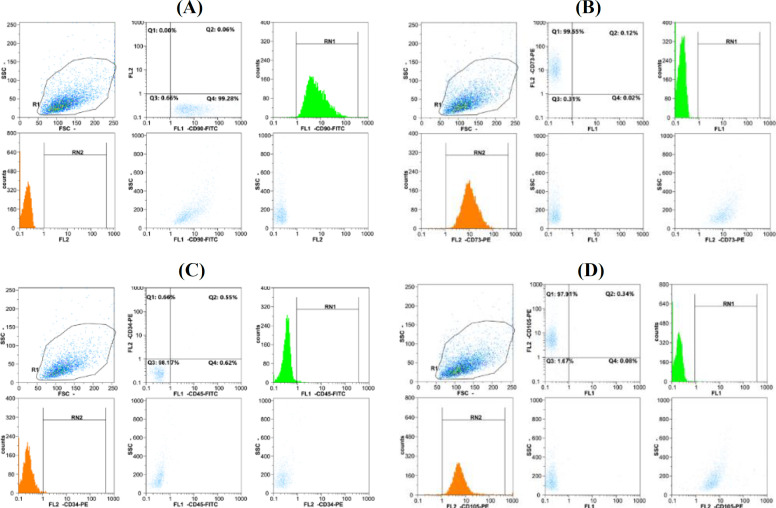
Identification of hUCMSCs. The surface markers CD105, CD34, CD45, CD73, and CD90 were determined by flow cytometry assay. The results demonstrated that cells were positive for (A) CD90 (99.28%), (B) CD73 (99.55%), (D) CD105 (97.91%), and negative for (C) CD45 and CD34

The hUCMSCs were evaluated after the sixth passage by phase contrast microscopy to examine the morphology. The hUCMSCs had a spindle-shaped morphology, which adhered to plastic culture wells or flasks. The results of immunophenotyping demonstrated that the cells were positive for CD90, CD105, and CD73 and negative for CD45 and CD34 (Fig. 1), confirming that these cells were human umbilical cord-blood-derived MSCs.

**Verification of hUCMSC-MVs using TEM, DLS, and Bradford methods**

**Fig. 2:**
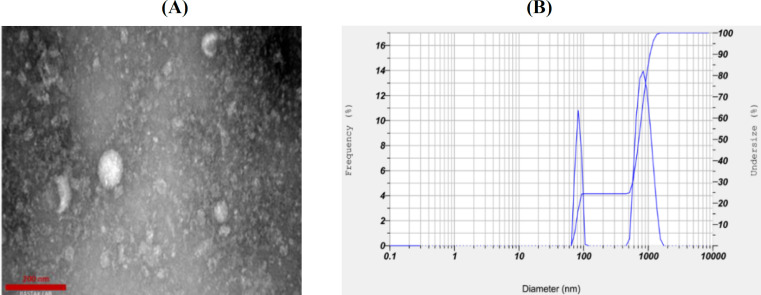
Identification of hUCMSC-MVs. (A) Morphology of hUCMSC-MVs under a transmission electron microscope (scale bar: 200 nm); (B) representative of MV size distribution histogram obtained from DLS analysis

**Fig. 3:**
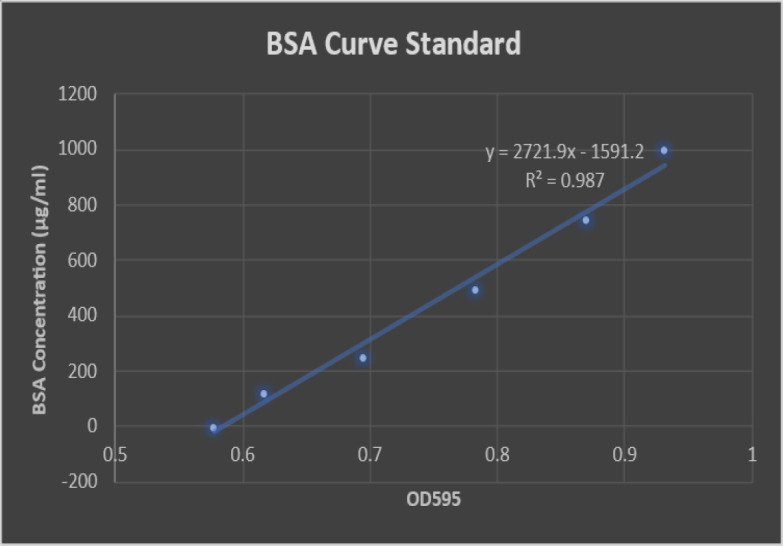
The protein content of MVs, which was 114.52 µg/ml

The hUCMSC-MVs were isolated and purified from the supernatant from the hUCMSCs using ultracentrifugation and then characterized by TEM. The TEM result showed that the size of MVs was approximately between 100 to 1000 nm (Fig. 2). MVs were heterogeneous lipid bilayer vesicles characterized as cup-shaped or irregular-shaped (Fig. 2A). The isolated hUCMSC-MVs were also characterized by DLS to study their size using the analyzer HORIBA SZ-100A2 (HORIBA, Japan). The particles had an average diameter of 643.5 nm with two distinct peaks at 78.4 and 825.9 nm, which confirmed that the isolated particles were MVs (Fig. 2B). Standard curve was plotted using sequential dilutions from a specific concentration of bovine serum albumin protein. The content of protein in the isolated MVs was 114.52 μg/ml, as shown in Figure 3.

**Apoptosis evaluation of KG-1 cells after treatment with hUCMSC-MVs** **in vitro**

**Fig. 4:**
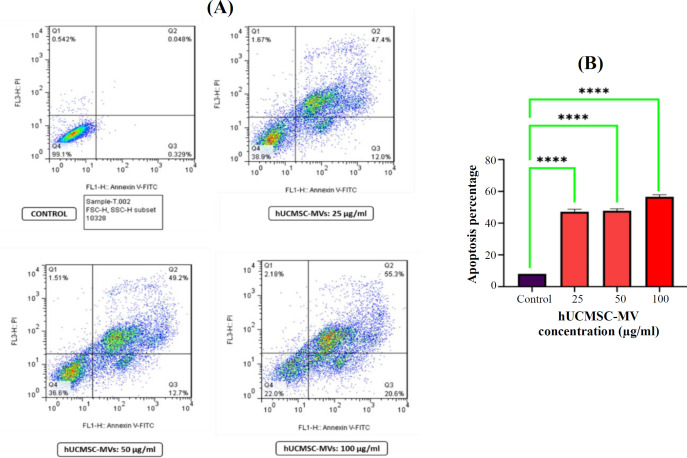
Effect of hUCMSC-MVs on apoptosis of KG-1 cells. (A) Flowcytometric analysis of annexin-V/PI staining of KG-1 after treatment with 25, 50, 100 µg/mL of MSC-MVs and control sample for 24 h. The Figure represents the percentage of early (annexin-V+, PI-) and late (Annexin-V+, PI+) apoptotic cells of leukemic cells, as well as the percentage of (annexin-V-, PI+) necrosis cells and live cells (annexin-V-, PI-); (B) Apoptosis analysis of different concentrations of hUCMSC-MVs compared to the control samples. All three concentrations of MVs were replicated three times. (^****^p ≤ 0.0001).

To determine the apoptotic effect of hUCMSC-MVs on KG-1 cells, we evaluated the cell death using annexin V-FITC/PI staining. The results showed that the percentage of early apoptotic cells treated with 25, 50, and 100 μg/ml of hUCMSC-MVs for 24 h was 12%, 12.7%, and 20.6%, respectively, and that of the control cells was 0.329%. Also, the percentages of the population of late apoptotic cells treated with 25, 50, and 100 μg/ml were 47.4%, 49.2%, and 55.3%, respectively,

whereas the control sample had 0.048% of late apoptotic cells (Fig. 4). The three treated samples showed a significant increase compared to the control sample (p < 0.0001). 

**Intracellular ROS accumulation in KG-1 cells after treatment with hUCMSC-MVs** **in vitro**

**Fig. 5:**
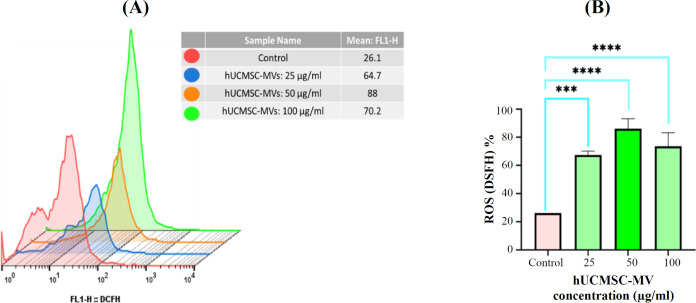
Intracellular ROS levels after hUCMSC-MVs treatment in KG-1 cells. (A) Flow cytometric analysis determined ROS activity of KG-1 after treatment with 25, 50, 100 µg/mL of MSC-MVs and control sample for 24 h. The expression level of DCFH (FL1-H) is shown in the table, and the number reported indicate mean: FL1-H (FL1-H: intracellular ROS accumulation level). (B) ROS activity of KG-1 cells promoted by treatment with hUCMSC-derived MVs. Three concentrations of MSC-MVs were replicated three times (^***^p ≤ 0.001 and ^****^p ≤ 0.0001).

To determine the effect of hUCMSC-MVs on intracellular ROS accumulation in KG-1 cells, DCFH_2_-DA was used (Fig. 5). The results revealed that intracellular ROS level at all three concentrations of 25, 50, and 100 μg/ml was 64.7% (p < 0.0002), 88% (p < 0.0001), and 70.2% (p < 0.0001), respectively, compared to the control group. The hUCMSC-MVs at 50 μg/ml had the most significant (p < 0.0001) effect on increasing the ROS levels.

**Expression of proapoptotic and antiapoptotic genes**

**Fig. 6:**
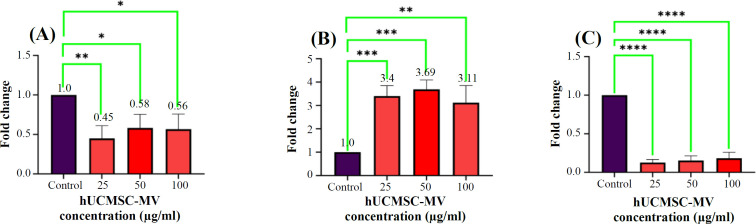
Expression levels of *BCL-2* (A) and *BAX* (B) genes in KG-1 cells after treatment with hUCMSC-MVs at concentrations of 25, 50 and 100 μg/ml compared to the control sample by RT-PCR. (C) Comparison of *BCL-2*/*BAX* gene expression in KG-1 cells after treatment with hUCMSC-MVs at concentrations of 25, 50 and 100 μg/ml (^****^p < 0.0001). All three concentrations of huCMSC-MVs in both genes were replicated three times ([A] ^*^p < 0.05, ^**^p < 0.001; [B] ^**^p < 0.0.01, ^***^p < 0.0001).

Expression of *BAX* (proapoptotic protein) and *BCL-2* (prosurvival protein) genes involved in apoptosis was examined in KG-1 cells after treating the KG-1 cells with hUCMSC-MV for 24 h. Following the treatment, the mRNA expression of *BAX* was almost 3.4-fold (p < 0.0007), 3.69-fold (p < 0.0007), and 3.11-fold (p < 0.0015) at 25, 50, and 100 μg/ml concentrations of huCMSC-MVs, respectively, indicating a significant increase at three concentrations compared to the control samples. However, the mRNA expression of *BCL-2* was approximately 0.45-fold (p < 0.005), 0.58-fold, and 0.56-fold (p < 0.02) at 25, 50, and 100 μg/ml concentrations of hUCMSC-MVs, demonstrating a significant decrease at three concentrations compared to the control samples. In addition to the mentioned genes, the ratio of *Bcl-2*/*BAX* is a common method to assess the level of apoptosis. Our data showed that this ratio decreased significantly (p < 0.0001) in the three study groups treated with different conc entrations of hUCMSC-MVs (Fig. 6).

**Expression of autophagy genes**

**Fig. 7:**
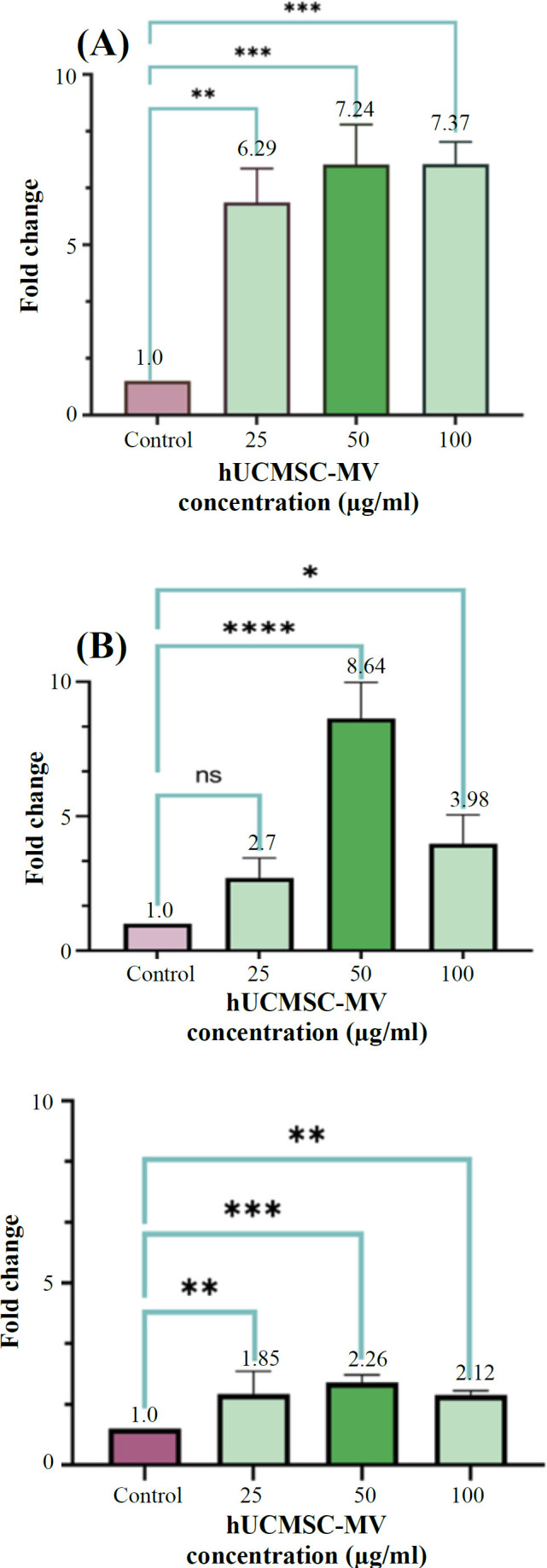
Expression levels of *Beclin 1* (A), *LC3* (B), and (C) *ATG7* genes in KG-1 cells after treatment with hUCMSC-MVs at concentrations of 25, 50, and 100 μg/ml compared to the control sample by RT-PCR. All three concentrations of hUCMSC-MVs in both genes were replicated three times ([A] ^**^p < 0.001, ^***^p < 0.0009; [B] ^*^p < 0.0112, ^****^p < 0.0001; [C] ^**^p < 0.0042, ^***^p < 0.0009).

The expression of *ATG7*, *LC3*, and *Beclin 1* autophagy genes was examined in KG-1 cells after hUCMSC-MV treatment for 24 h. Our findings showed that the expression of *ATG7* was almost 1.85-fold (p < 0.0042), 2.26-fold (p < 0.0009), and 2.12-fold (p < 0.0042) at 25, 50, and 100 μg/ml concentrations of hUCMSC-MV, respectively. The expression of *LC3* was ~2.7-fold at the concentration of 25 μg/ml, which was not significant compared to the control sample, 8.64-fold at the concentration of 50 μg/ml (p < 0.0001), and 3.98-fold at a concentration of 100 μg/ml (p < 0.0112). Furthermore, the *Beclin 1* expression was 6.29-fold (p < 0.001), 7.24-fold (p < 0.0009), and 7.37-fold (p < 0.0009) higher at 25, 50, and 100 μg/ml concentrations, respectively compared to the control sample, which was significantly enhanced at all three concentrations. In addition, 50 μg/ml of hUCMSC-MVs had the most significant effect on *LC3* (p < 0.0001) and *ATG7* (p < 0.0009) expression (Fig. 7). 

## DISCUSSION

Currently, cell-free therapies are considered attractive and promising approaches for melanoma, as well as colorectal, non-small cell lung carcinoma, human papilloma virus-induced, and non-virus-induced cancers^[^^23^^,^^24^^]^. Clinically, the use of cell-derived particles, such as MSC-MVs, in transferring bioactive molecules has strong advantages over traditional interventions. Unlike MSCs, MSC-MVs are safer and easier to store and handle. MSC-MVs carry several types of molecules, including proteins/peptides, mRNAs, microRNAs, and lipids, which induce intercellular communication and have potential tumor-suppressive properties^[^^25^^-^^27^^]^. Studies have reported that MVs inhibit the growth, proliferation, and progression of the cell cycle in tumor cells and increase apoptosis in cancer cells^[^^14^^,^^25^^]^. In our study, MVs were successfully isolated from the supernatant of hUCMSCs, and the results indicated that MVs could induce apoptosis and autophagy in the KG-1 cells. Similarly, Phetfong et al.^[^^28^^]^ showed that MSC extracellular vesicles increased the expression of *BID* and *BAX* and decreased the expression of *BCL2* in NB4 cells, as well as increased the expression of the death receptor gene *TRAILR2* and cell cycle regulator genes *P21* and *CCNE2* in K562 cells.

AML is a common blood cancer with serious morbidity and high mortality rates. However, effective therapies, mechanisms of progression, diagnostic biomarkers, and prognosis of AML remain obscure^[^^29^^]^. Moreover, it is not clear whether MSC-EVs have an inhibitory or stimulatory effect on cancer progression in terms of type of cancer cells or not. We, in this study, investigated the role of MVs derived from hUCMSCs in the apoptotic and autophagic pathways of the leukemic cell line (KG-1). MVs increased apoptotic cells significantly after treatment with three concentrations of hUCMSC-MVs as compared to the control sample (p < 0.0001). The mean percentages of late apoptotic cells at 25, 50, and 100 µg/ml concentrations of hUCMSC-MVs were 47.85%, 47.15%, and 51.35%, respectively, compared to 0.048% in the control sample. In addition, the mean percentages of living cells at three mentioned concentrations were 39.55%, 37.85%, and 21.2%, respectively, in a concentration-dependent manner. 

Our apoptosis findings were in line with previous studies^[^^14^^,^^17^^,^^25^^,^^30^^]^. Wu et al.^[^^14^^]^ demonstrated that treatment of T-24 cells with MVs of hWJMSC-MVs increased apoptosis both in vivo and in vitro, and hWJMSC-MVs exerted potent antiproliferative and proapoptotic effects on bladder tumor T24 cells. In another study, Zhang et al.^[^^31^^]^ found low cell viability rate, high apoptosis ratio, and low IRF1/INPP4B expression in THP-1 cells exposed to exosomes derived from human bone marrow MSCs. Besides a significant increase in apoptotic cells, RT-PCR results of the present study suggested that *BCL-2* gene expression in KG-1 cells treated with hUCMSC-MVs significantly reduced compared to the control group, and *BAX* gene expression as a proapoptotic protein significantly increased, with the highest expression of *BAX* at a concentration of 50 μg/ml. The *BCL-2*/*BAX* ratio, an important indicator of the apoptotic process decreased in every three hUMSC-MVs concentrations. In this regard, Ji et al.^[^^17^^]^ suggested that by treatment of K562 and HL-60 cell lines with MVs released from the MSCs derived from human embryonic stem cells, MVs, the *BCL-2*/*BAX* ratio decreased at the concentrations of 30 (p < 0.05) and 60 µg/ml (p < 0.01), which were similar to our results. In addition, bone marrow MSC-EVs increased the expression of *BID* and *BAX* and decreased the *BCL2* expression, indicating the induction of intrinsic apoptosis in the NB4 cell line. Multiple studies have indicated that following the co-culture of ovarian cancer cell line ES-2 (clear cell carcinoma) and OAW-42 (cystadenocarcinoma) with MVs derived from human immortalized MSCs, originating from adipose

\

tissue, the average percentage of apoptotic cancer cells increased, while the percentage of live cancer cells decreased^[^^32^^,^^33^^]^. Similarly, bone marrow MSCs-derived exosomes effectively suppressed cell proliferation (at 10 and 20 mg/ml of exosome) and cell cycle progression at G0-G1 stage, as well as significantly enhanced cell apoptosis in KG-1a cells^[^^34^^]^.

ROS accumulation and autophagy-related gene expression were examined to evaluate autophagy in cells treated with MVs. The measurement of intracellular ROS levels in KG-1 cells treated with MVs enhanced compared to that of the control sample, which may promote autophagy. It is noteworthy that 50 μg/ml of hUCMSC-MVs had the greatest effect on ROS increase in KG-1 cells (p < 0.0001). Analysis of autophagy-associated gene expression of Beclin1, LC3, and ATG7 showed increased expression of these genes in cells treated with MVs compared to the control sample. In this regard, Ji et al.^[^^17^^]^ reported that the co-culture of K562 and HL60 cells with MVs induced autophagosomes and electron-dense vacuoles containing degraded organelles and increased the levels of *Beclin 1* and LC3-II formation, an indicator of autophagic activity, compared to untreated groups. Chen et al.^[^^35^^]^ also reported that hWJMSC-MVs enhanced autophagy and ameliorated acute lung injury via the delivery of miR-100, which was comparable to our results.

Overall, the present study suggests that hUCMSC-MVs may play a critical role in autophagy induction in KG-1 cells and promote apoptosis, similar to the results of previous studies showing the antiproliferative and pro-apoptotic effects of MSC-EVs on leukemic cells (NB4, KG-1a, and K562)^[^^31^^,^^34^^,^^37^^]^. The hUCMSC-MVs could inhibit the growth of leukemic cells, in which either autophagy or apoptosis might be the responsible mechanism, indicating that MVs have a high potential in AML therapy. However, there are still many challenges in the clinical application of hUCMSC-MVs for the treatment of AML.

## DECLARATIONS

### Acknowledgments

 The authors acknowledge the financial support of this study by the Department of Hematology and Blood Transfusion Sciences, Tehran University of Medical Sciences, Tehran, Iran.

### Ethical statement

 The present study was approved by the Committee on Ethics of the Tehran University of Medical Sciences, Tehran, Iran (ethical code: IR.TUMS.SPH.REC.1399. 159).

### Data availability

The analyzed data sets generated during the study are available from the corresponding author on reasonable request.

### Author contributions

MKE: study conception and design, acquisition of data, analysis and interpretation of data, wrote the paper, and review and editing; SHM: study conception and design, analysis and interpretation of data, review and editing; MZ: study conception and design; JMK: acquisition of data, analysis and interpretation of data; ZZB: wrote the paper and review and editing.

### Conflict of interest

 None declared.

### Funding/support

This study was financially supported by the Tehran University of Medical Sciences Foundation, Tehran, Iran (grant number: 95-01-31-31461).
